# An Extensive Replication Study on Three New Susceptibility Loci of Primary Angle Closure Glaucoma in Han Chinese: Jiangsu Eye Study

**DOI:** 10.1155/2013/641596

**Published:** 2013-10-24

**Authors:** Haihong Shi, Rongrong Zhu, Nan Hu, Jian Shi, Junfang Zhang, Linjuan Jiang, Hong Jiang, Huaijin Guan

**Affiliations:** Eye Institute, Affiliated Hospital of Nantong University, 20 Xisi Road, Nantong 226001, Jiangsu, China

## Abstract

Genome-wide association study (GWAS) analysis identified three new susceptibility loci for PACG. In this study, we aimed to investigate whether these three loci in PLEKHA7, COL11A1, and PCMTD1-ST18 are associated with PAC and ocular biometric characteristics, such as axial length (AL), anterior chamber depth (ACD), and diopter of spherical power (DS). The study was a part of the Jiangsu Eye Study. The samples were collected from 232 PAC subjects and 306 controls from a population-based prevalence survey conducted in Funing County of Jiangsu, China. The single nucleotide polymorphisms (SNPs) of rs11024102 in PLEKHA7, rs3753841 in COL11A1, and rs1015213 in PCMTD1-ST18 were genotyped by TaqMan-MGB probe using the RT-PCR system. None of the three polymorphisms showed differences in the distribution of genotypes and allele frequencies between the PAC group and the control group. No significant association was determined between the 3 SNPs and AL, ACD, or DS of PAC subjects. We concluded that even though PLEKHA7 rs11024102, COL11A1 rs3753841, and PCMTD1-ST18 rs1015213 are associated with PACG, those sequence variations are not associated with PAC in a Han Chinese population. Our results also did not support a significant role for these three SNPs in ocular biometry such as AL, ACD, and DS.

## 1. Introduction

Glaucoma is the second leading cause of irreversible blindness worldwide. Clinically, primary glaucoma presents two major subtypes: primary open-angle glaucoma (POAG) and primary angle closure glaucoma (PACG). The classification relies mainly on the anterior segment anatomy, particularly that of the anterior chamber angle. PACG is characterized by obstruction of aqueous fluid drainage through the trabecular meshwork from the anterior chamber of the eye. The anterior chamber depth (ACD) is a main factor affecting the drainage of aqueous humor. PACG affects as many as 4.5 million people in China, and it has been reported that Asian populations are at higher risk of developing PACG than other ethnic groups [1]. 

Eyes with PACG usually display characteristic anatomical features such as a shorter corneal diameter, a steeper corneal curvature, a shallower anterior chamber, a thicker and more anteriorly positioned lens, and a shortened eyeball, often accompanied by hyperopic refraction error [2]. The risk factors for developing PACG include age, family history, and being female [3]. First-degree relatives were found to have a 6- to 9-fold increased risk of developing PACG [4]. Siblings of Chinese patients with PAC or PACG have almost a 50% probability of having narrow angles and are more than 7 times more likely to have narrow angles than the general population [5]. Ethnic differences are also associated with PACG. There is also a higher prevalence among Inuits and Asians compared to Caucasians, suggesting a genetic predisposition for the disorder [6].

Because the ocular anatomic features are predisposing factors for PACG, genes involved in regulation of axial length and structural remodeling of connective tissues may contribute to development of PACG. Some tissue remodeling genes including membrane frizzled-related protein (MFRP) [7, 8], extracellular matrix metalloprotease-9 (MMP-9) [9–11], and methylenetetrahydrofolate reductase (MTHFR) [12] have been reported to be associated with PACG. Even though heat shock protein 70 (HSP70) does not regulate tissue remolding directly, it regulates the expression of matrix metalloproteases (MMPs) and is thought to be associated with PACG [13]. However, the above findings remain controversial and have not been replicated by independent studies. 

Recently, a genome-wide association study (GWAS) identified three new susceptibility loci for PACG, including rs11024102 in PLEKHA7, rs3753841 in COL11A1, and rs1015213 in PCMTD1-ST18 [14]. However, the mechanism of these genes in PACG pathogenesis is unclear, and the biological plausibility is absent. We hypothesized that PLEKHA7, COL11A1, and PCMTD1-ST18 might contribute to PACG by influencing ocular biometry. Thus, in this study, we attempted to replicate the association between these three loci with primary angle closure (PAC) instead of PACG to investigate whether the SNPs of these three genes are associated with ocular biometry. PAC is the earlier stage of PACG and shares the same anatomical features; however, PAC does not present glaucomatous optic neuropathy. Our definition of PAC includes asymptomatic individuals with occludable angles who have not had an acute attack as well as those who have had an attack but received prompt treatment and suffered no detectable nerve damage.

## 2. Methods 

### 2.1. Study Subjects

The study was a part of the Jiangsu Eye Study and was conducted according to the Declaration of Helsinki and approved by the Ethics Committee of the Affiliated Hospital of Nantong University. Each participant was fully informed of the purpose and procedures involved in the study and signed the Informed Consent Form. The general demographic information of the participants is listed in Table 1. All participants were recruited from a population-based prevalence survey on eye diseases using a cluster random sampling strategy in Funing County of Jiangsu, China. Of the 6032 people screened, 232 people with PAC and 306 controls were enrolled in the study. PAC subjects and controls were matched in groups for sex and age and were ethnically homogenous. The participants were unrelated and self-identified Han Chinese. There was no difference between the control group and the PAC group in gender, age, or systemic disease distribution.

All study participants were residents of Funing County of Jiangsu, China, aged 50 years and above. Each participant received a thorough ophthalmic examination, included best-corrected visual acuity, anterior segment photography, Goldmann applanation tonometry, fundus examination, optic disc photography, visual field, objective refraction, and subjective refraction. The depth of the peripheral anterior chamber was determined using Van Herick technique [15]. The subjects with a peripheral chamber depth less than one-third of corneal thickness were invited for gonioscopy, A-scan ultrasonography, and ultrasound biomicroscopy (UBM, SW-3200S, SUOER, China) examinations. UBM examinations were conducted in light and dark conditions in eight positions. The detailed protocol for gonioscopy and UBM was reported previously by Barkana et al. [16]. Three measurements of ACD and AL were made by A-scan to get mean values, and mean values of binoculus were used for statistical analyses.

PAC was defined according to the International Society of Geographical and Epidemiologic Ophthalmology (ISGEO) classification by Foster et al. [17]: (1) either eye has the presence of an occluded angle (at least 180 degrees of closed angle in which the trabecular meshwork is not visible on gonioscopy or iris apposition to the trabecular meshwork more than 180 degrees on UBM); (2) at least one of the following features was detected: peripheral anterior synechiae, intraocular pressure >21 mmHg, excessive pigment deposition on the superior trabecular meshwork, iris whirling, history of symptoms, or intraocular pressure elevated ≥8 mmHg after UBM examination in dark conditions; (3) no signs of secondary angle closure; (4) no signs of glaucomatous optic neuropathy and peripheral visual loss; (5) no previous ocular surgery or laser therapy. The clinical features of the PAC subjects are listed in Table 2.

The criteria for enrollment of the control group were (1) peripheral chamber depth more than one-third of corneal thickness, (2) intraocular pressure less than 21 mmHg, (3) normal optic nerve heads with cup-to-cup ratio less than 0.5, (4) normal visual field, (5) no family history of glaucoma, (6) no ophthalmic diseases except slight cataract, and (7): refractive error less than three diopters.

### 2.2. SNP Genotyping

Genomic DNA was extracted from the peripheral blood of each individual using the Qiagen Blood DNA Mini Kit (Qiagen, Valencia, CA), according to the manufacturer's instructions and stored at −20°C.

The samples were genotyped by TaqMan Aenotyping Assay (Applied Biosystems, Foster City, CA, USA) using the Real-time PCR 7500 system (Applied Biosystems, Foster City, CA, USA). The assay IDs are C_2981015_10 for rs11024102 (an SNP in intron region), C_2947954_10 for rs3753841 (a missense SNP), and C_7479939_10 for rs1015213 (a SNP in intergenic region). PCR reactions were performed in a total volume of 10 *µ*L containing 1 *µ*L (10 ng) DNA, 5 *µ*L TaqMan Universal Master Mix, 0.20 *µ*L TaqMan SNP Genotyping Assay Mix (40x), and 3.8 *µ*L Dnase-free, sterile filtered water. Amplification was carried out with an initial denaturation at 95°C for 5 min, followed by 40 cycles of denaturation at 95°C for 30 s and annealing at 60°C for 30 s.

### 2.3. Statistical Analysis

Statistical analysis was performed with SPSS version 15.0 software. Differences in age and gender between PAC subjects and controls were assessed using *t*-test and Chi-Square test, respectively. Hardy-Weinberg equilibrium was tested using Chi-Square test. To analyze the association of these three SNPs with PAC and controls, the frequency of genotypes and alleles were evaluated using Chi-Square test. *P* values < 0.05 were considered statistically significant. Logistic regression analysis was performed to calculate the odds ratio (OR) value, the 95% confidence interval (95% CI), and to adjust the confounding effects of age and gender. If any positive association was found in the initial analysis, Bonferroni correction was performed. Three genetic models were analyzed: the additive model defined as minor allele homozygotes versus heterozygotes versus common allele homozygotes, the dominant model as heterozygotes plus minor allele homozygotes versus common allele homozygotes, and the recessive model as minor allele homozygotes versus common allele homozygotes plus heterozygotes. The association of these three SNPs with AL, ACD, and DS was also assessed under the additive genetic model, dominant model, and recessive model using *t*-test.

## 3. Results

The call rates of all SNP genotyping were 100% and the call accuracies were 100% in a randomly selected 10% sample. All 3 SNPs conformed to Hardy-Weinberg equilibrium (*P* > 0.05) except for PCMTD1-ST18 rs1015213 in controls.

None of the three polymorphisms showed differences in the distribution of allele frequencies (Table 3) and genotypes (Table 4) between the cases and controls.

All three SNPs were not significantly associated with biometric parameters including ACD, AL, and DS (Table 5).

## 4. Discussion

This study, to the best of our knowledge, is the first population-based study to investigate the association of rs11024102, rs3753841, and rs1015213 with PAC and PAC relevant biometric parameters such as ACD, AL, and DS in a Han Chinese population. The design of a population-based study can minimize sample selection bias often present in hospital-based case-control study. We attempted to replicate the association between these three loci with PAC instead of PACG to verify the relationship between these SNPs and anatomic features. The results show that the variations of PLEKHA7 rs11024102, COL11A1 rs3753841, and PCMTD1-ST18 rs1015213 were not associated with either PAC or biometric factors in Han Chinese population. 

PLEKHA7 encodes pleckstrin homology domain-containing protein 7, which is involved in the maintenance and stability of epithelial and endothelial adherens junctions [18]. PLEKHA7 is expressed in the cornea, iris, and trabecular meshwork (TM). Increased resistance to drainage of aqueous humor through the pressure-dependent TM is believed to be responsible for POAG [19]. However, the pathogenesis of PACG is distinct from that of POAG. Eyes with PACG tend to share certain anatomic biometric characteristics and have nothing to do with aqueous humor outflow facility. In our present study, we did not find any association between rs11024102 and PAC, nor did we find any association between rs11024102 and biometric parameters.

COL11A1 gene codes for one of the two *α*-chains of type XI collagens. Type XI collagen is a minor fibril-forming collagen, controlling fibril growth, diameter, and assembly of major collagens. It is expressed primarily in the articular cartilage and the ocular vitreous [20]. Mutations in COL11A1 cause Marshall syndrome, Stickler syndrome, and Stickler-like syndrome; these disorders are all characterized by midfacial hypoplasia, sensorineural hearing deficit, and nonprogressive axial myopia [21]. Hyperopic and shorter axial length, but not axial myopia, is well-known predisposing factor for PACG. In our present study, the distribution of genotypes of rs3753841 was similar in the PAC and in the control group, as were the biometric parameters.

Rs1015213 is located upstream of PCMTD1 and downstream of ST18. PCMTD1 encodes protein-l-isoaspartate O-methyltransferase domain-containing protein 1 that is expressed in the cornea, iris, and TM. ST18 encodes the suppression of tumorigenicity 18 protein, expressed in the cornea and lens, but not in the TM [14]. In our study, the minor allele frequency of rs1015213 was low, which is consistent with previous reports [14, 22]. Little is known about the function of PCMTD1 or ST-18. There was no significant difference between the two groups in the genotype frequency or alleles for rs1015213 nor any significant difference between rs1015213 and biometric parameters.

Our results were not in line with Vithana et al.'s report [14] that reported the three loci susceptible for PACG by a GWAS study with a two-stage strategy. Sample size and ethnic distribution are two main factors that can influence the results of genotype association studies. Vithnan's study included 1854 PACG cases from an Asian population in stage 1 and 1917 PACG cases from 6 sample collections (two in China, and one each in UK, Singapore, India, and Saudi Arabia). The power analysis based on their data indicated that our study is underpowered (<50%) to detect any association of the 3 tested SNPs. However, all subjects included in this study are Han Chinese and subjects in both groups were age and gender matched. Moreover, the study was community based, thus decreasing the confounding of possible population stratification. We believe that our sample size is reasonable to detect a biologically meaningful association if it exists.

Another possible reason that we did not replicate the Vithana's report might be due to the different definition of the phenotypes, PAC in our study and PACG in Vithana's study. Because the number of PACG patients in this community cohort did not meet the basic requirements to conduct an independent association study, we excluded this phenotype. Day et al. [22] conducted a genotype-phenotype analysis of these three SNPs with the ocular biometry of 988 European people. They found that the A allele of rs1015213 was nominally associated with ACD (*P* = 0.046) but not associated with AL or corneal keratometry. Rs11024102 and rs1015213 were not associated with ocular biometry, which is consistent with our results. 

Another limitation in our study is that AL and ACD parameters are only available for the PAC group. It is time consuming and technically demanding to invite all 6032 participants for UBM, gonioscopy, and A-scan examinations. Additionally, the development of PACG is complex and likely depends on polygenic inheritance. It appears that each anatomic characteristic is not determined by a series of independent genes acting with no relation to other components but is instead an additive outcome of the action of a large number of genes. The effect of each gene would be small and difficult to distinguish individually. 

## 5. Conclusion

The sequence variants of PLEKHA7 rs11024102, COL11A1 rs3753841, and PCMTD1-ST18 rs1015213 do not appear to be associated with PAC and ocular biometry in our study. Because the PLEKHA7 rs11024102, COL11A1 rs3753841, and PCMTD1-ST18 rs1015213 were reported to be associated with PACG, the lack of association of these SNPs may be due to a different phenotype being assessed.

## Figures and Tables

**Table 1:** Demographics of study participants.

Demographic features	Control *n* (%)	PAC *n* (%)	*P*
Female	248 (81.05)	191 (82.33)	0.70
Male	58 (18.95)	41 (17.67)	
Mean age (year) ± SD	65.08 ± 7.53	64.84 ± 8.59	0.74
Age range	50–85	50–83	
Hypertension	66 (19.64)	46 (19.83)	0.69
Diabetes	24 (7.36)	20 (8.6)	0.76
Cardiovascular	10 (3.27)	4 (1.72)	0.41

**Table 2:** Clinical features of PAC subjects.

	Right eye (mean ± SD)	Left eye (mean ± SD)	Mean of both eyes (mean ± SD)
Axial length (mm)	22.17 ± 0.83	22.17 ± 0.82	22.17 ± 0.83
ACD (mm)	2.49 ± 0.29	2.45 ± 0.30	2.47 ± 0.29
Refractive (diopter)	0.53 ± 1.85	0.68 ± 1.87	0.58 ± 1.84
Tonometry (mmHg)	15.18 ± 4.31	15.78 ± 4.46	15.52 ± 4.39

**Table 3:** Allele frequency of SNPs in control and PAC subjects.

SNP	Allele distribution/minor/major (minor %)		*P*	OR (95% CI)
	Control	PAC		
PLEKHA7 rs11024102 (T/C)	245/367 (40.0)	199/265 (42.9)	0.346	1.13 (0.88–1.44)
COL11A1 rs3753841 (A/G)	195/417 (31.9)	136/328 (29.3)	0.369	0.88 (0.68–1.15)
PCMTD1-ST18 rs1015213 (C/T)	13/599 (2.1)	11/453 (2.4)	0.786	1.12 (0.50–2.51)

All HWE *P*  values > 0.05 except for PCMTD1-ST18 in controls.

**Table 4:** Genotype frequency of SNPs in control and PAC subjects.

SNP	Genotype distribution *n* (%)			General *P* value	Dominant p/OR (95% CI)	Recessive p/OR (95% CI)
		Control	PAC			
PLEKHA7 rs11024102 (T/C)	TT	105 (34.3)	78 (33.6)	0.283	0.87/1.03 (0.72–1.48)	0.12/1.43 (0.91–2.26)
	TC	157 (51.3)	109 (47.0)			
	CC	44 (14.4)	45 (14.4)			
COL11A1 rs3753841 (A/G)	AA	145 (47.4)	116 (19.4)	0.606	0.55/0.90 (0.64–1.27)	0.34/0.75 (0.42–1.40)
	AG	127 (41.5)	96 (41.4)			
	GG	34 (11.1)	20 (8.6)			
PCMTD1-ST18 rs1015213 (C/T)	CC	295 (96.4)	221 (95.3)	0.261	0.51/1.34 (0.56–3.14)	0.51/0.26 (0.01–5.49)
	CT	9 (2.9)	11 (4.7)			
	TT	2 (0.6)	0 (0.0)			

**Table 5:** The relationship of biometric parameters with genotypes of rs1015213, rs375384, and rs11024102 in PAC group.

	Genotype	AL (mm) (mean ± SD)	ACD (mm) (mean ± SD)	Refrative power (D) (mean ± SD)
PLEKHA7 rs11024102	TT	22.16 ± 0.70	2.44 ± 0.23	0.64 ± 1.29
	TC + CC	22.15 ± 0.76	2.47 ± 0.22	0.74 ± 1.63
*P*		0.958	0.448	0.663

COL11A1 rs3753841	AA	22.11 ± 0.72	2.46 ± 0.23	0.71 ± 1.46
	AG + GG	22.20 ± 0.76	2.46 ± 0.22	0.70 ± 1.58
*P*		0.366	0.924	0.945

PCMTD1-ST18 rs1015213	CC	22.15 ± 0.72	2.46 ± 0.22	0.70 ± 1.55
	CT + TT	22.29 ± 0.99	2.42 ± 0.27	0.80 ± 0.68
*P*		0.528	0.617	0.835
